# 基于分子分型的广泛期小细胞肺癌免疫治疗专家共识

**DOI:** 10.3779/j.issn.1009-3419.2026.102.18

**Published:** 2026-07-20

**Authors:** 中国临床肿瘤学会肿瘤生物标志物专家委员会 浙江省抗癌协会肿瘤呼吸病学专业委员会

**Keywords:** 肺肿瘤, 分子分型, 免疫治疗, 病理检测, 专家共识, Lung neoplasms, Molecular subtyping, Immunotherapy, Pathological testing, Expert consensus

## Abstract

免疫检查点抑制剂联合铂类-依托泊苷化疗已成为广泛期小细胞肺癌（extensive-stage small cell lung cancer, ES-SCLC）的一线标准治疗方案。然而患者的治疗获益差异显著，长期生存率仍然有限。近年来，基于ASCL1、NEUROD1和POU2F3转录因子的表达特征以及免疫炎症状态建立的SCLC-A、SCLC-N、SCLC-P和SCLC-I分型体系，为解释ES-SCLC免疫治疗异质性和开展精准分层治疗提供新的依据。为规范分子分型在ES-SCLC免疫治疗中的临床转化，本共识组织呼吸内科、肿瘤内科、病理科、放疗科及基础医学等多学科专家，基于国内外循证医学证据和临床经验，围绕ES-SCLC一线免疫治疗现状、分子分型体系及免疫微环境特征、检测实施路径、免疫治疗相关生物标志物、分型治疗策略关键问题形成推荐意见。本共识强调，分子分型现阶段应定位为标准治疗基础上的生物学和临床研究分层工具，不宜作为独立治疗决策依据；免疫组化可作为具备条件中心开展分型探索和转化研究的重要方法之一；SCLC-I亚型可作为免疫治疗潜在获益人群的重要生物标志物之一；针对不同分型，delta样配体3（delta-like ligand 3, DLL3）靶向治疗、表观遗传调控、MYC/AURKA轴以及聚腺苷二磷酸核糖聚合酶（poly(ADP-ribose) polymerase, PARP）抑制剂可作为后续治疗优化的重要探索方向。本共识旨在推动ES-SCLC分子分型病理检测与临床应用的规范化，促进基于分子分型的免疫治疗分层管理和个体化治疗策略优化，为构建ES-SCLC精准免疫治疗体系提供依据。

## Abstract (en)

Immune checkpoint inhibitors combined with Platinum-based Etopositde chemotherapy have become the first-line standard treatment for extensive-stage small cell lung cancer (ES-SCLC). However, treatment benefit varies substantially among patients, and the proportion of long-term survivors remains limited. In recent years, the molecular classification framework comprising SCLC-A, SCLC-N, SCLC-P and SCLC-I, based on the expression patterns of ASCL1, NEUROD1 and POU2F3 as well as immune-inflammatory status, has provided a new basis for understanding the heterogeneity of immunotherapy response and for developing precision stratified treatment in ES-SCLC. To standardize the clinical translation of molecular subtyping in ES-SCLC immunotherapy, this consensus was developed by multidisciplinary experts from respiratory medicine, oncology, pathology, radiation oncology, and basic medical sciences. Based on domestic and international evidence and clinical experience, the consensus provides recommendations on key issues, including first-line immunotherapy for ES-SCLC, molecular subtyping systems and immune microenvironment characteristics, testing and implementation pathways, immunotherapy-related biomarkers, subtype-informed treatment strategies, and dynamic monitoring. This consensus emphasizes that molecular subtyping should currently be positioned as a tool for biological and clinical research stratification based on standard treatment, and should not be used as an independent basis for treatment decisions. Immunohistochemistry can serve as an important method for qualified centers to conduct subtyping exploration and translational research. The SCLC-I subtype may provide a useful reference for biomarker-based stratification and immunotherapy benefit prediction. For different subtypes, delta-like ligand 3 (DLL3)-targeted therapy, epigenetic regulation, the MYC/AURKA axis, and poly (ADP-ribose) polymerase (PARP) inhibitors can be important choices for subsequent treatment optimization. This consensus aims to standardize pathological testing and clinical application of molecular subtyping in ES-SCLC, facilitate stratified management and individualized treatment optimization based on molecular subtypes, and provide a foundation for building a precision immunotherapy system.

肺癌是全球死亡率最高的恶性肿瘤^[1]^，小细胞肺癌（small cell lung cancer, SCLC）是一种神经内分泌（neuroendocrine, NE）肿瘤，占肺癌的13%-15%。SCLC生长速度快、恶性程度高、肿瘤转移早，约2/3的患者在诊断时为广泛期SCLC（extensive-stage SCLC, ES-SCLC），5年生存率低于7%^[2,3]^。数十年来，依托泊苷联合铂类化疗是ES-SCLC一线标准治疗方案，尽管初期疗效显著，但绝大多数患者短期内复发，中位总生存期（overall survival, OS）难以突破1年，临床面临巨大挑战。

免疫检查点抑制剂（immune checkpoint inhibitors, ICIs）的出现为ES-SCLC的治疗带来了历史性突破。IMpower133与CASPIAN等关键III期研究^[4,5]^相继证实，一线治疗中在依托泊苷联合铂类化疗方案基础上联合程序性死亡配体1（programmed death-ligand 1, PD-L1）抑制剂，能显著延长患者的OS，由此确立了免疫治疗联合化疗作为ES-SCLC一线新标准治疗的地位。此后，多种程序性死亡受体1（programmed cell death protein 1, PD-1）/PD-L1抑制剂（如阿得贝利单抗、斯鲁利单抗、特瑞普利单抗、替雷利珠单抗等）陆续在中国获批，为临床提供了更多选择。值得一提的是，ETER701研究^[6]^探索了PD-L1抑制剂贝莫苏拜单抗联合抗血管生成药物安罗替尼及化疗的疗效，将中位OS提升至19.3个月，创造了ES-SCLC一线治疗生存期的新纪录。尤为引人关注的是，对IMpower133的长期随访分析^[7]^显示，接受阿替利珠单抗联合化疗方案治疗的患者，其5年OS率达12%，这为ES-SCLC的长期生存带来了新的希望。

然而，尽管免疫治疗联合化疗为部分ES-SCLC患者带来了生存希望，但其临床获益存在显著异质性，中位OS仅小幅提升约2个月，且仅在15%-20%的患者中观察到持久应答^[8]^。这种疗效差异与SCLC高度的肿瘤异质性密切相关，目前研究结果^[9,10]^已证实，传统标志物如肿瘤突变负荷（tumor mutational burden, TMB）和PD-L1在SCLC免疫治疗疗效中的预测效果不理想。因此，寻找能够精准识别获益人群的新型生物标志物，已成为当前领域破局的关键。

近年来，SCLC分子分型研究为精准免疫治疗提供了新方向。基于4种关键转录因子achaete-scute家族bHLH转录因子1（achaete-scute family bHLH transcription factor 1, ASCL1）、神经元分化因子1（neuronal differentiation 1, NEUROD1）、POU结构域2类转录因子3（POU class 2 homeobox 3, POU2F3）和Yes1相关转录调控因子（Yes1 associated transcriptional regulator, YAP1），将SCLC划分为SCLC-A（ASCL1主导）、SCLC-N（NEUROD1主导）、SCLC-P（POU2F3主导）和SCLC-Y（YAP1主导）4种亚型^[11]^。后续研究^[12]^进一步鉴定出一个以高表达抗原呈递相关基因和T细胞免疫特征为标志的“炎症”亚型（SCLC-inflamed, SCLC-I）。对IMpower133的回顾性分析^[13]^显示，SCLC-I亚型患者接受阿替利珠单抗联合化疗的中位OS达18.2个月，显著优于化疗组的10.4个月，提示SCLC-I亚型患者可能从免疫治疗联合化疗中获得更显著的生存改善。

本共识旨在总结近年来ES-SCLC分子分型与免疫治疗的研究进展，提出基于分型的免疫治疗分层管理建议，推动分子分型在临床决策中的规范化应用和推广，进而提升ES-SCLC精准诊疗水平，促进个体化治疗策略的优化与临床转化。

## 1 方法学

### 1.1 共识机构和专家组成员

本共识由中国临床肿瘤学会（Chinese Society of Clinical Oncology, CSCO）肿瘤生物标志物专家委员会和浙江省抗癌协会肿瘤呼吸病学专业委员会发起，联合国内呼吸内科、肿瘤内科、病理科、肺癌综合诊治科、放疗科、基础医学等多学科专家组成的专家组牵头制定。共识制定工作自2026年1月启动，首先，由牵头专家组结合ES-SCLC免疫治疗临床实践中的关键问题，确定共识主题及核心临床问题；随后，围绕ES-SCLC一线免疫治疗现状、分子分型体系、肿瘤免疫微环境（tumor immune microenvironment, TIME）特征、检测实施路径、基于分子分型的治疗策略等内容开展文献检索、证据整理和质量评估，并由执笔组根据现有证据和临床实践经验起草共识初稿及推荐意见。

共识初稿形成后，专家组经过2轮线上投票表决。第一轮和第二轮问卷有效回收率均为100%。第一轮投票围绕共识框架、核心临床问题及17条推荐意见初稿进行评议，并收集专家对推荐意见表述、证据依据、推荐强度及临床适用性的修改建议。第一轮投票后，17条推荐意见均达到预设共识标准。对于专家提出实质性修改意见或需进一步明确表述的条目，执笔组根据反馈意见进行了文字修订、证据补充和推荐强度核对，并将修订后的共识意见3、4、5、6、8、9、12和14提交第二轮投票复评；复投条目均达到预设共识标准。所有参与专家均对本共识内容、推荐意见及证据等级进行了审阅和确认，并声明不存在与本共识制定相关的利益冲突。

### 1.2 文献检索

共识根据ES-SCLC免疫治疗的临床问题设置关键检索词，并通过不同平台检索引擎的筛选功能，检索不同的研究类型文献。检索数据库主要为PubMed、Embase、国际大会官网、中国知网和万方数据知识服务平台，具体检索策略采用以下关键词组合：“small cell lung cancer（SCLC）或small cell carcinoma”和“immunotherapy或immune checkpoint inhibitors（ICIs）或programmed death receptor 1（PD-1）或programmed death-ligand 1（PD-L1）”和“molecular subtypes”；中文检索关键词以“小细胞肺癌”和“分子分型”和“免疫治疗/免疫检查点抑制剂”为主。

文献纳入类型包括国内外指南、共识、随机对照试验、系统性综述、荟萃分析、前瞻性队列研究、回顾性队列研究、单臂临床研究和基础转化探索研究等。检索时限为建库至2026年3月31日。

### 1.3 证据等级评定标准和推荐强度

本共识推荐意见按推荐等级和证据级别分别进行评估，见表1。推荐强度与证据等级的划分参考国内已发表肺癌专家共识的分级方法^[14]^。

**表1:** 推荐强度和证据等级标准

推荐强度和证据级别	标准
推荐强度	
强（80%-100%）	绝大多数专家达成共识
中（60%-79%）	多数专家达成共识但少数专家存在分歧
弱（50%-59%）	专家未达成一致性结论
未达成共识（<50%）	原则上不形成正式推荐意见，需进一步讨论、修改或暂不纳入
证据等级	
A（1类）	严谨的meta分析、大型随机对照研究
B（2类）	一般质量的meta分析、小型随机对照研究、设计良好的大型回顾性研究、病例-对照研究
C（3类）	非对照的单臂临床研究、病例报告、临床前研究、专家观点

需要说明的是，本共识中证据等级与推荐强度反映不同维度。证据等级主要依据现有文献类型和证据质量划分，推荐强度主要依据专家组投票赞同率，反映专家对推荐意见的共识程度。因此，部分C级证据条目若专家一致性较高，仍可形成强推荐，但其临床应用仍需结合证据局限和现阶段转化研究属性谨慎解释。

### 1.4 使用人群

本共识可供肿瘤内科、呼吸内科、病理科、放疗科及多学科团队等医疗专业人士使用。

## 2 ES-SCLC一线免疫治疗现状与临床困境

多项关键III期研究证实，在铂类-依托泊苷化疗基础上联合PD-L1/PD-1抑制剂，可为ES-SCLC患者带来生存获益，并由此奠定了当前一线标准治疗模式（表2^[4,6,7,15-23]^）。IMpower133研究评估了阿替利珠单抗联合依托泊苷+卡铂（Etoposide plus Carboplatin, EC）化疗方案在ES-SCLC一线治疗中的疗效。对比安慰剂联合EC方案，阿替利珠单抗联合EC方案可显著延长患者的OS和无进展生存期（progression-free survival, PFS），中位OS分别为12.3和10.3个月（HR=0.70, 95%CI: 0.54-0.91, P=0.007），中位PFS分别为5.2和4.3个月（HR=0.77, 95%CI: 0.62-0.96, P=0.02）^[4]^，5年OS率为12%，优于历史上单纯化疗的5年OS率（约2%）^[7]^。2020年2月国家药品监督管理局（National Medical Products Administration, NMPA）批准阿替利珠单抗联合EC方案用于ES-SCLC患者一线治疗。

**表2:** PD-1/PD-L1抑制剂联合化疗用于ES-SCLC一线治疗的III期临床研究

药物类型及研究名称	治疗方案	n	ORR	mPFS（月）	mOS（月）	2年OS率	3年OS率
PD-L1单抗							
IMpower133^[4,7]^	阿替利珠单抗+依托泊苷+卡铂	201	60.2%	5.2	12.3	22%	16%
	安慰剂+依托泊苷+卡铂	202	64.4%	4.3	10.3	16%	NE
CASPIAN^[15,16]^	度伐利尤单抗+依托泊苷+卡铂/顺铂	268	68.0%	5.1	12.9	22.9%	17.6%
	度伐利尤单抗+曲美木单抗+依托泊苷+卡铂/顺铂	268	58.0%	4.9	10.4	22.9%	15.3%
	依托泊苷+卡铂/顺铂	269	58.0%	5.4	10.5	13.9%	5.8%
CAPSTONE-1^[17,18]^	阿得贝利单抗+依托泊苷+卡铂	230	70.4%	5.8	15.3	30.9%	21.1%
	安慰剂+依托泊苷+卡铂	232	65.9%	5.6	12.8	17.8%	10.5%
ETER701^[6]^	贝莫苏拜单抗+安罗替尼+依托泊苷+卡铂	246	81.3%	6.9	19.3	/	/
	安慰剂+安罗替尼+依托泊苷+卡铂	245	81.2%	5.6	13.3	/	/
	安慰剂+安慰剂+依托泊苷+卡铂	247	66.8%	4.2	11.9	/	/
NCT04878016^[19]^	索卡佐利单抗+依托泊苷+卡铂	250	75.5%	5.55	13.90	20.7%	/
	安慰剂+依托泊苷+卡铂	248	68.1%	4.37	11.58	5.9%	/
PD-1单抗							
ASTRUM-005^[20]^	斯鲁利单抗+依托泊苷+卡铂	389	68.9%	5.8	15.8	31.7%	/
	安慰剂+依托泊苷+卡铂	196	58.7%	4.3	11.1	18.7%	/
EXTENTORCH^[21]^	特瑞普利单抗+依托泊苷+卡铂/顺铂	223	78.0%	5.8	14.6	25.9%	/
	安慰剂+依托泊苷+卡铂/顺铂	219	73.1%	5.6	13.3	19.5%	/
RATIONALE-312^[22,23]^	替雷利珠单抗+依托泊苷+卡铂/顺铂	227	68%	4.7	15.5	33.1%	22.1%
	安慰剂+依托泊苷+卡铂/顺铂	230	62%	4.3	13.5	22.4%	13.1%

表内数据为不同研究结果的描述性汇总，因研究设计与随访条件不同，不宜直接横向比较。IMpower133主要分析^[4]^报告ORR、PFS及OS；5年随访^[7]^报告2及3年OS率，对照组3年OS率不可估（未转入IMbrella A）。CASPIAN更新分析^[15]^报告ORR、PFS、OS及2年OS率；3年随访^[16]^报告3年OS率。CAPSTONE-1主要分析^[17]^报告ORR、PFS及OS；3年更新^[18]^（会议摘要）报告2及3年OS率。ETER701^[6]^报告ORR、PFS及OS。NCT04878016^[19]^、ASTRUM-005^[20]^及EXTENTORCH^[21]^报告ORR、PFS、OS及2年OS率。RATIONALE-312主要分析^[22]^报告ORR、PFS、OS及2年OS率；长期随访^[23]^报告3年OS率。PD-1：程序性死亡受体1；PD-L1：程序性死亡配体1；ES-SCLC：广泛期小细胞肺癌；ORR：客观缓解率；mPFS：中位无进展生存期；mOS：中位总生存期。NE为不可评估，/为无数据。

CASPIAN研究^[5]^评估了度伐利尤单抗联合含铂化疗方案在ES-SCLC一线治疗中的疗效。结果显示，度伐利尤单抗联合依托泊苷+顺铂（Etoposide plus Cisplatin, EP）或EC化疗组中位OS为13.0个月，单纯依托泊苷联合铂类（EC/EP）化疗组为10.3个月（HR=0.73, 95%CI: 0.59-0.91, P=0.0047）。度伐利尤单抗联合化疗组2年OS率为22.9%，3年OS率为17.6%，而单纯化疗组分别为13.9%和5.8%^[15,16]^。2021年7月，NMPA批准度伐利尤单抗联合EC/EP方案用于ES-SCLC患者一线治疗。IMpower133和CASPIAN两项关键研究共同奠定了ICIs联合铂类-依托泊苷化疗作为ES-SCLC一线标准治疗地位的循证医学基础。

在此基础上，多项后续的III期研究，尤其是中国人群主导的注册研究，进一步拓展并丰富了这一治疗框架。CAPSTONE-1研究^[17]^评估了阿得贝利单抗联合EC化疗方案在ES-SCLC一线治疗中的疗效，与EC化疗联合安慰剂组相比，EC化疗联合阿得贝利单抗显著延长了患者的OS，中位OS分别为15.3和12.8个月（HR=0.72, P=0.0017），其长期随访数据（会议摘要）显示，3年OS率分别为21.1%和10.5%^[18]^。2023年3月，NMPA批准阿得贝利单抗联合EC方案用于ES-SCLC的一线治疗。

ETER701研究^[6]^探索了PD-L1抑制剂贝莫苏拜单抗联合抗血管生成药物安罗替尼及化疗的疗效，与EC化疗组相比，贝莫苏拜单抗与安罗替尼联合EC组显著延长了患者的中位OS（19.3 vs 11.9个月，HR=0.61）和PFS（6.9 vs 4.2个月，HR=0.32）。2024年5月，NMPA批准贝莫苏拜单抗联合安罗替尼、EC方案用于ES-SCLC的一线治疗。

此外，NCT04878016研究^[19]^评估了索卡佐利单抗联合EC方案用于ES-SCLC一线治疗的疗效。与EC方案化疗组相比，索卡佐利单抗联合EC方案能够延长患者的OS和PFS，中位OS分别为13.90和11.58个月（HR=0.799, P=0.0158），中位PFS分别为5.55和4.37个月。2025年7月，NMPA批准索卡佐利单抗联合EC方案用于ES-SCLC的一线治疗。

ASTRUM-005研究^[24]^证实了PD-1抑制剂斯鲁利单抗联合EC方案在ES-SCLC一线治疗中的疗效。延长随访分析^[20]^提示，安慰剂联合EC化疗组的中位PFS和OS分别为4.3和11.1个月，而斯鲁利单抗联合EC化疗组的中位PFS和OS分别为5.8和15.8个月；2年OS率分别为18.7%和31.7%。2023年1月，NMPA批准斯鲁利单抗联合EC方案用于ES-SCLC患者的一线治疗。

EXTENTORCH研究^[21]^评估了特瑞普利单抗联合化疗对比安慰剂联合化疗在ES-SCLC一线治疗中的疗效。结果显示，特瑞普利单抗联合化疗可延长患者OS和PFS（中位OS：14.6 vs 13.3个月，HR=0.8，P=0.03；中位PFS：5.8 vs 5.6个月，HR=0.67，P<0.001）。2024年6月，NMPA批准特瑞普利单抗联合EC/EP方案用于ES-SCLC患者的一线治疗。

RATIONALE-312研究^[22]^评估了替雷利珠单抗联合化疗用于ES-SCLC一线治疗的安全性和有效性。替雷利珠单抗联合化疗组的PFS和OS较化疗组延长，中位PFS分别为4.7和4.3个月（HR=0.64, P<0.0001），OS分别为15.5和13.5个月（HR=0.75, P=0.004）。其长期随访数据显示，3年OS率分别为22.1%和13.1%^[23]^。2024年6月，NMPA批准替雷利珠单抗联合EC方案用于ES-SCLC的一线治疗。基于这些研究结果，上述多种PD-1/PD-L1抑制剂联合EC/EP方案已陆续在中国获批用于ES-SCLC的一线治疗。

然而，必须看到，即便在免疫治疗联合化疗时代，ES-SCLC患者总体预后仍不理想，且不同患者之间的疗效差异显著，提示该疾病存在高度生物学异质性。目前PD-L1表达和TMB在ES-SCLC一线免疫治疗联合化疗中的预测价值尚未得到稳定验证，不建议作为常规筛选获益人群或决定是否采用一线免疫治疗联合化疗的依据^[9,10]^。正是在这一背景下，分子分型的临床意义逐渐凸显。但是目前有关分型与免疫治疗疗效关系的证据，主要来自探索性转化分析、回顾性研究及临床试验的生物标志物亚组研究，尚缺乏基于前瞻性分型驱动设计的随机临床试验证据。因此，分子分型在现阶段的价值，应定位于优化风险分层、揭示免疫治疗异质性、辅助临床研究分层及推动新策略开发，而非直接替代现有标准一线治疗决策。

**专家共识1：对于初诊ES-SCLC患者，一线治疗仍推荐ICIs联合铂类-依托泊苷化疗为标准方案（证据等级：A，推荐强度：强）。**

**专家共识2：ES-SCLC分子分型应定位为标准治疗基础上的生物学分层工具与精准治疗增益策略，而非替代一线免疫联合化疗标准方案的独立决策依据（证据等级：A，推荐强度：强）。**

**专家共识3：目前PD-L1表达和TMB水平在ES-SCLC免疫治疗中的预测价值尚不明确，不建议将其作为筛选免疫治疗获益人群或决定是否采用一线免疫治疗联合化疗的依据；分子分型可用于生物学分层和临床研究分层（证据等级：B，推荐强度：强）。**

## 3 ES-SCLC分子分型体系及其免疫学特征

### 3.1 ES-SCLC分子分型的定义基础及主要生物学特征

近年来，基于转录组学的分子分型研究揭示了SCLC的高度异质性。目前国际学术界已初步形成共识，采用以3个关键谱系转录因子（ASCL1、NEUROD1、POU2F3）的相对表达水平及1个独特的免疫炎症特征为基础的分子分型概念，即SCLC-A、SCLC-N、SCLC-P、SCLC-I分型。Rudin等^[11]^在2019年首次系统性地提出了基于关键转录因子ASCL1、NEUROD1、POU2F3和YAP1的分子分型框架，为SCLC的异质性研究奠定了理论基础。在此基础上，Gay等^[12]^通过对肿瘤转录组数据的非负矩阵分解（non-negative matrix factorization, NMF）分析，确立了SCLC-A、SCLC-N、SCLC-P、SCLC-I分子分型系统，其中SCLC-I亚型被定义为不依赖前述转录因子，但具有独特免疫炎症特征的群体，该研究同时证实SCLC-I亚型患者能够从ICIs联合化疗中显著获益。

SCLC-A型（ASCL1驱动）是SCLC中最常见的亚型，占SCLC的36%-78%^[11,12,25,26]^。该亚型具有典型的NE特征，高表达胰岛素瘤相关蛋白1（insulinoma-associated protein 1, INSM1）、嗜铬粒蛋白A（chromogranin A, CgA）和突触素（synaptophysin, SYP）等NE标志物。其分子特征包括肺癌衍生v-myc骨髓细胞瘤病毒癌基因同源物（v-myc avian myelocytomatosis viral oncogene lung carcinoma derived homolog, MCYL）扩增，以及delta样配体3（delta-like ligand 3, DLL3）和B细胞淋巴瘤2（B-cell lymphoma 2, BCL-2）的高表达^[11]^，并富集细胞周期进程与DNA损伤修复（DNA damage repair, DDR）相关通路基因^[27]^，而NOTCH通路通常处于抑制状态^[28]^。

SCLC-N型（NEUROD1驱动）占SCLC的11%-31%^[11,12,25,26]^，同样为NE高表达亚型。其特征为MYC扩增和激活，Aurora激酶A（Aurora kinase A, AURKA）活性增高^[29]^，并表现出更强的转移倾向^[27]^。

SCLC-P型（POU2F3驱动）占SCLC的3%-16%^[11,12,25,26]^，属于非神经内分泌（non-NE）亚型。其关键驱动因子POU2F3是肺上皮簇细胞关键谱系调控因子，该亚型因此也被称为簇细胞样亚型；SRY相关高迁移率族盒蛋白9（SRY-related high-mobility group box 9, SOX9）、ASCL2和胰岛素样生长因子1受体（insulin-like growth factor 1 receptor, IGF1R）是该亚型的关键下游因子^[30]^。

SCLC-I型（免疫炎症型）占7%-18%^[12,25,26]^，其特征为ASCL1、NEUROD1及POU2F3均低表达。该亚型表现出上皮间质转化和显著的炎症特征，高表达人类白细胞抗原（human leukocyte antigen, HLA）、γ干扰素（interferon-γ, IFN-γ）激活以及免疫检查点相关基因^[12]^。

SCLC-A、SCLC-N、SCLC-P、SCLC-I分型更适宜理解为由优势转录程序主导的生物学状态，而非绝对互斥、静态不变的固定亚型；在治疗压力等因素影响下，不同亚型之间还可能发生动态转化。现有证据^[25,31]^表明，YAP1在初治SCLC中普遍呈低表达，且在初治肿瘤组织与动物模型中难以界定稳定的YAP1高表达群体。其高表达多为疾病复发或治疗耐药后出现的动态表型，与获得性耐药有关^[32,33]^。病理学研究^[25,34]^进一步证实，YAP1表达主要见于复合型SCLC，并常与ASCL1、NEUROD1等已知亚型标志物共表达，不具备独立性。基于上述证据，本共识认为，YAP1高表达代表了一种由肿瘤可塑性驱动的获得性表型状态，而非独立的驱动亚型。

### 3.2 ES-SCLC不同分子分型的TIME特征

SCLC虽然具有较高的TMB，但其TIME普遍存在免疫细胞浸润不足、抗原呈递功能低下等特征，整体上被认为是一种免疫抑制性肿瘤^[35]^。分子分型研究^[36]^进一步揭示了不同亚型TIME的内在异质性，并明确了其与ICIs疗效的关联。总体而言，SCLC-A与SCLC-N型（NE高表达亚型）多呈现“免疫荒漠”表型，而SCLC-P及SCLC-I（non-NE亚型）则倾向于呈现“免疫炎症”表型，后者与更好的PD-1/PD-L1抑制剂疗效相关。

SCLC-A型表现为典型的“免疫荒漠”表型。该亚型肿瘤主要组织相容性复合体I/II类分子（major histocompatibility complex class I/II, MHC-I/II）表达水平低，抗原呈递能力弱^[37]^；其TIME中CD8^+^效应T细胞（effector T cells, T-eff）浸润稀少，而调节性T细胞（regulatory T cells, Tregs）与耗竭T细胞相对富集，形成免疫抑制状态^[38]^。在表观遗传层面，组蛋白甲基转移酶zeste增强子同源物2（enhancer of zeste homolog 2, EZH2）不仅参与维持ASCL1的表达，还能直接抑制MHC-I的表达，从而阻碍抗原呈递，抑制自然杀伤（natural killer, NK）细胞激活所需的配体（如NKG2DL），并且抑制负责招募抗原呈递细胞和T细胞的趋化因子（如CCL2）^[39]^。

SCLC-N型同样表现为“免疫荒漠”表型，且免疫抑制程度更为显著。该亚型在所有亚型中免疫相关基因表达水平最低，其T细胞组成高度失调，表现为Tregs和耗竭CD8^+^ T细胞显著升高，而CD8^+^ T-eff和γδT细胞比例显著降低^[26,27]^。此外，SCLC-N与促纤维化、具有免疫抑制功能的单核/巨噬细胞群体相关，进一步强化了其免疫抑制性微环境^[27]^。

SCLC-P型具有较高的CD8^+^ T细胞和NK细胞浸润，肿瘤及间质中PD-L1与MHC-I/II表达水平升高，并且肿瘤中三级淋巴结构密度较高，呈现“炎症型”免疫表型^[26,40,41]^。但是，该亚型中FOXP3^+^ Tregs显著增多，且与CD8^+^ T细胞及NK细胞的相互作用增强，提示其虽具有较活跃的免疫微环境，但同时伴随明显的免疫抑制特征^[42]^。

SCLC-I型（免疫炎症型）是典型的免疫“热肿瘤”。该亚型表现出显著的T细胞（尤其是细胞毒性T细胞）浸润，并伴有NK细胞、巨噬细胞等多种免疫细胞数量的显著增加。其TIME具有完整的抗原呈递机制（如HLA高表达）和活跃的干扰素信号通路。同时，该亚型高表达PD-1/PD-L1、细胞毒性T淋巴细胞相关抗原4（cytotoxic T-lymphocyte-associated protein 4, CTLA-4）及其配体、含Ig和ITIM结构域的T细胞免疫受体（T cell immunoreceptor with Ig and ITIM domains, TIGIT）、淋巴细胞活化基因-3（lymphocyte activation gene-3, LAG-3）等多种免疫检查点分子，且干扰素基因刺激因子（stimulator of interferon genes, STING）通路下游的T细胞趋化因子CCL5与CXCL10也显著高表达，共同塑造了一个适于ICIs发挥作用的炎症微环境^[12]^。

**专家共识4：建议参考采用以ASCL1、NEUROD1、POU2F3表达特征及免疫炎症状态为基础的SCLC-A、SCLC-N、SCLC-P、SCLC-I分型框架，用于ES-SCLC免疫治疗获益人群识别、临床研究分层及治疗策略优化（证据等级：B，推荐强度：强）。**

## 4 ES-SCLC分子分型及相关生物标志物检测与实施建议

### 4.1 ES-SCLC分子分型检测方法与规范化实施

ES-SCLC分子分型最初建立在转录组学研究基础上。Rudin团队^[11]^根据81例SCLC临床肿瘤样本RNA测序数据将SCLC分成SCLC-A、SCLC-N、SCLC-P和SCLC-Y亚型，占比分别为70%、11%、16%和2%。随后，Gay等^[12]^通过NMF对上述81例SCLC的RNA数据进行分析，鉴定出SCLC-A、SCLC-N、SCLC-P和SCLC-I 4种亚型，占比分别为36%、31%、16%和17%。上述结果提示，SCLC分子分型在一定程度上受分析方法和分型算法影响，不同研究间亚型构成比例可存在差异。

在临床实践中，尽管转录组检测能够较为全面地反映肿瘤的分子特征，但受限于检测成本、技术平台和样本质控等因素，其常规推广仍存在一定限制性。相比之下，免疫组化（immunohistochemistry, IHC）因其可及性强、流程成熟、成本相对可控等优势，是目前最具现实推广价值的分型实施路径。目前已有研究^[31]^证实，采用IHC检测ASCL1、NEUROD1和POU2F3蛋白表达水平进行分子分型，与基于全转录组测序及NMF的分型结果具有高度一致性。尽管不能直接替代转录组层面的SCLC-I分型，IHC仍有助于识别具有炎性免疫特征的TIME。

在病理结果具体判读方法上，大部分的研究采用IHC评分H-score系统（即阳性肿瘤细胞百分比与染色强度的乘积）进行IHC定量^[26]^。对于SCLC-A、SCLC-N和SCLC-P亚型，通常可根据ASCL1、NEUROD1和POU2F3 3种关键转录因子的相对表达水平进行判定，并以H-score最高者定义优势亚型；必要时亦可结合聚类分析等方法进行进一步归类。与此不同，SCLC-I亚型目前尚缺乏统一、公认的单一特异性IHC标志物，其病理判定更强调在ASCL1、NEUROD1和POU2F3均低表达（H-score评分<10）或阴性的基础上，结合TIME相关IHC特征进行综合评估^[26]^。现有研究^[43]^提示，SCLC-I样肿瘤常表现为较高密度的CD8^+^肿瘤浸润淋巴细胞，可通过CD8 IHC染色进行辅助识别。此外，IHC检测揭示了ES-SCLC存在显著的瘤内异质性。研究^[26]^显示，17.6%的肿瘤同时表达2种亚型标志物，2.8%同时表达3种。ASCL1/NEUROD1共表达型（SCLC-A/N）占17.9%，其基因表达谱和临床结局更接近SCLC-A而非SCLC-N^[31]^，提示IHC不仅可用于优势亚型判定，也有助于识别混合表达和过渡状态，从而更真实地反映SCLC的生物学复杂性。此外，SCLC-I亚型的判读不应简单等同于ASCL1、NEUROD1和POU2F3 3种转录因子低表达或阴性。3种转录因子阴性结果需首先排除样本质量、抗体保存、染色平台及判读误差等技术因素。在具备可靠质控的基础上，SCLC-I亚型应结合免疫炎症相关特征进行综合判断。因此对于仅表现为3种转录因子低表达而缺乏明确炎症微环境证据的病例，应谨慎解释，避免将技术性假阴性误判为SCLC-I亚型。

值得注意的是临床诊断中可获得的ES-SCLC组织多为支气管镜或计算机断层扫描（computed tomography, CT）引导下肺穿刺活检等小标本，因此小标本条件下分型检测的可行性尤为关键。研究^[44,45]^显示，无论是组织微阵列（tissue microarray, TMA）样本与手术切除标本之间，还是肺穿刺活检标本与手术切除标本之间，亚型特异性标志物IHC检测阳性判定均具有高度一致性，表明活检小样本可用于SCLC分子分型的IHC检测。但是需要强调的是，部分小标本可存在肿瘤组织量有限、挤压伪影、坏死比例较高、固定不充分及抗原保存不佳等问题。因此，开展基于IHC的分子分型检测时，应首先评估标本内外部质控、广谱上皮标志物如细胞角蛋白（cytokeratin, CK）染色表现、肿瘤细胞量、坏死比例、挤压伪影及标本制备质量。若存在CK染色不支持足够肿瘤成分、存在明显挤压伪影、广泛坏死或固定不充分的标本，不建议强行进行分型判读，可报告为“分型结果不确定”或“暂不适合分型评估”，必要时建议重新取材、补充活检或结合转录组等研究性检测进一步确认。

基于现有证据，本共识建议各病理实验室积极开展ES-SCLC分子分型的IHC检测并逐步规范检测流程，统一标本固定、取材和染色等前处理要求，合理选择抗体克隆、检测平台及配套质控体系，并结合H-score等半定量方法完善判读标准。在结果判读和报告中，除明确优势亚型外，还应重视低表达、双标志物共表达及多标志物共表达等情况，避免对存在异质性的肿瘤进行过度简化归类。总体而言，ES-SCLC分子分型的IHC检测具有较好的临床可实施性和推广前景，未来应在标准化、同质化和临床验证持续推进的基础上，逐步推动其在精准诊疗中的规范应用。

### 4.2 ES-SCLC免疫相关生物标志物的检测及建议

除肿瘤细胞内在分型外，TIME特征同样可能影响ES-SCLC对免疫治疗的敏感性。CASPIAN研究^[46]^分析表明，较高的CD8^+^ T细胞浸润和MHC-I/II相关分子表达与ICIs治疗的最大生存获益相关。CheckMate 032研究^[47]^也提示抗原加工与呈递机制（antigen-presenting and processing machinery, APM）基因谱高表达与纳武利尤单抗治疗的OS获益显著关联。同时T-eff与肿瘤相关巨噬细胞（tumor-associated macrophages, TAMs）平衡对免疫治疗疗效具有重要预测价值。对IMpower133试验的深入分析^[36]^显示，尽管SCLC-I-non-NE和SCLC-I-NE亚型均具有T细胞炎症特征，但前者因富含TAMs而获益有限（HR=1.02），而后者（T-eff-high/TAMs-low）从阿替利珠单抗治疗中获益显著（HR=0.45）。IFN-γ信号通路活性是识别长期获益患者的重要特征。空间转录组研究^[48]^发现，获得长期获益（PFS≥12个月）的患者肿瘤中普遍存在以IFN-γ信号为主导的信使RNA（messenger RNA, mRNA）表达谱，其特征是抗原呈递能力增强。

但需要强调的是，上述免疫功能标志物目前仍以探索性证据为主，尚未形成统一检测标准、通用阈值和前瞻性验证体系。故本共识更倾向于将其定位为有条件开展的转化研究检测项目，用于临床试验分层、生物标志物探索及机制研究，而非现阶段所有ES-SCLC患者的常规必检指标。

### 4.3 ES-SCLC分子分型的前沿检测方法

液体活检具有微创、便于重复采样的优势，能有效克服肿瘤异质性，实现对治疗疗效与耐药性的实时动态监测。循环游离DNA（circulating free DNA, cfDNA）的甲基化特征谱不仅可用于SCLC的分子分型，还可追踪疾病进展中的动态演化^[49]^。此外循环外泌体中ASCL1、NEUROD1、POU2F3关键亚型定义转录因子mRNA的检测，为SCLC分子分型提供了新的无创手段^[50]^。

近年来，单细胞转录组、空间转录组及蛋白基因组学等新技术的发展，进一步推动了ES-SCLC分型研究的深入。上述技术有助于更深入地揭示ES-SCLC的生物学异质性、优势克隆特征、治疗应答差异及耐药演化规律，并为筛选潜在获益人群和发掘新的治疗靶点提供重要依据。然而，鉴于其检测成本较高、技术平台依赖性较强，且目前尚缺乏统一的标准化流程和临床验证体系，此类方法现阶段更适合作为科研和前瞻性探索工具，尚不建议纳入常规临床实施路径。

**专家共识5：鉴于IHC具有可及性高、技术流程成熟、成本相对可控等优势，建议在具备条件的中心将其作为ES-SCLC分子分型探索性临床应用和转化研究的重要检测方法，并在不断完善规范化质控、判读标准和临床验证的基础上逐步推广（证据等级：C，推荐强度：强）。**

**专家共识6：建议由病理科或具备相应资质的检测实验室负责ES-SCLC分子分型检测实施与质量管控，规范标本制备、抗体选择、染色平台、质量控制及结果判读等关键环节，提高检测准确性与可重复性（证据等级：C，推荐强度：强）。**

**专家共识7：建议逐步建立并统一ES-SCLC分子分型IHC判读标准与报告规范；在明确优势亚型的同时，关注低表达、双/多标志物共表达等肿瘤异质性，以提升检测结果的可比性与临床解释力（证据等级：C，推荐强度：强）。**

**专家共识8：鉴于免疫治疗相关生物标志物，如CD8** ^**+**^ **T细胞浸润、MHC-I/II及抗原加工递呈相关分子、IFN-γ炎症基因特征、TAMs丰度、T-eff等，其预测价值、检测方法及判定阈值尚未统一，建议主要用于临床研究和转化探索，暂不列为临床常规必检项目；液体活检、单细胞测序及空间组学等技术可作为动态监测与机制研究的探索性工具，现阶段亦不建议常规临床开展（证据等级：C，推荐强度：强）。**

## 5 ES-SCLC分子分型与免疫治疗策略

现有证据表明，分子分型有助于理解ES-SCLC患者免疫治疗疗效差异背后的生物学基础，并可为联合治疗策略优化及临床研究分层提供参考（图1）。但应看到，分子分型目前尚不能单独作为治疗决策依据。除肿瘤内在亚型特征外，抗原递呈能力、CD8^+^ T细胞浸润、髓系TIME状态以及肿瘤细胞可塑性等因素，均可能影响患者对免疫治疗的实际反应。因此，在当前临床实践中，分型结果更适合作为标准治疗基础上的辅助参考，用于患者分层、优势人群识别及联合治疗探索，而不宜脱离临床特征、病理表现和治疗过程中的动态变化作决定性判断。

**图1:**
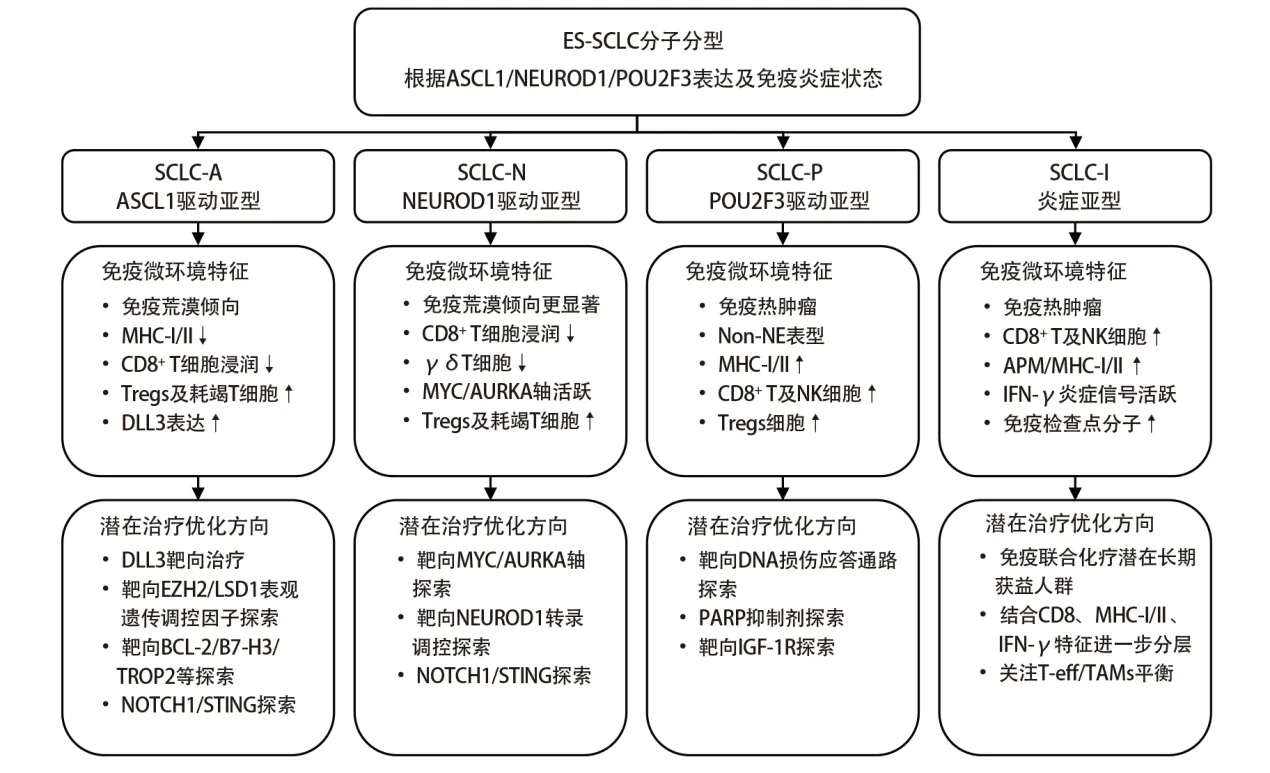
ES-SCLC分子亚型（SCLC-A/N/P/I）的免疫微环境特征及其潜在治疗策略 MHC：主要组织相容性复合体；Tregs：调节性T细胞；DLL3：delta样配体3；NK细胞：自然杀伤细胞；APM：抗原加工与呈递机制；IFN-γ：γ干扰素；EZH2：zeste增强子同源物2；LSD1：赖氨酸特异性脱甲基酶1；BCL-2：B细胞淋巴瘤2；B7-H3：B7同源物3；TROP2：滋养层细胞表面抗原2；STING：干扰素基因刺激因子；AURKA：Aurora激酶A；PARP：聚腺苷二磷酸核糖聚合酶；IGF-1R：胰岛素样生长因子1受体；T-eff：效应T细胞；TAMs：肿瘤相关巨噬细胞。

### 5.1 SCLC-I亚型

2021年Gay等^[12]^发表于Cancer Cell的奠基性研究，通过NMF将SCLC分为SCLC-A、SCLC-N、SCLC-P、SCLC-I 4个亚型。基于IMpower133试验数据的分析显示，SCLC-I亚型从免疫治疗联合化疗中获益最为显著，在化疗+阿替利珠单抗组中位OS超过18个月，而在化疗+安慰剂组仅为约10个月，表明SCLC-I可作为预测免疫治疗获益的生物标志物。尽管所有亚型均显示出从阿替利珠单抗中获益的趋势，但SCLC-I的获益幅度最大。值得注意的是，SCLC-P亚型在两组中均表现较差，提示其可能为不良预后因素。

IMpower133的长期随访^[7]^分析显示，阿替利珠单抗联合化疗的患者5年OS率可达12%，在这些患者中SCLC-A、SCLC-N、SCLC-I亚型均存在。另一项针对IMpower133研究的生物标志物分析^[13]^显示，接受阿替利珠单抗联合EC治疗的长期存活者（生存期≥18个月）中，SCLC-I亚型的比例（28%）显著高于非长期存活者（11%）。尤为重要的是，在全部SCLC-I亚型患者中，阿替利珠单抗组有55%实现长期生存，而单纯化疗组仅为30%。这表明SCLC-I亚型是预测从阿替利珠单抗联合化疗中获得长期生存获益的潜在预测性生物标志物。

Nabet等^[36]^对IMpower133研究进行了深入分析，在之前分型基础上揭示了SCLC炎症亚型内部的显著异质性。研究发现，具有免疫浸润特征的SCLC可被细分为2个亚型：SCLC-I-NE（保留NE特征）和SCLC-I-non-NE（呈non-NE表型）。疗效分析显示，阿替利珠单抗的显著生存获益主要局限于SCLC-I-NE亚型（中位OS：16.37 vs 8.63个月，HR=0.45），而在SCLC-I-non-NE亚型中未观察到显著优势（中位OS：9.19 vs 10.11个月，HR=1.02）。机制探索表明，SCLC-I-non-NE亚型尽管存在T细胞浸润，但其肿瘤微环境（tumor microenvironment, TME）中同时伴有显著的TAMs富集，形成了潜在的免疫抑制环境，从而抵消或削弱了PD-L1抑制剂的疗效，提示T细胞与TAMs的平衡与疗效有关。此外，non-NE表型的关键转录因子RE1沉默转录因子（RE1-silencing transcription factor, REST）的表达与髓系细胞趋化因子水平呈正相关，提示肿瘤细胞本身可能主动塑造了这种抑制性的微环境。

对CASPIAN的深入分析发现，SCLC-I亚型患者接受度伐利尤单抗±曲美木单抗（CTLA-4抑制剂）+化疗方案治疗后的中位OS最佳，达到24.0个月，显著高于SCLC-N（12.1个月）和SCLC-A（11.5个月）亚型，而SCLC-P亚型的预后最差，中位OS仅为7.0个月。该研究不仅验证了SCLC-I亚型是预测免疫化疗长期获益的有利指标，还发现CD8^+^ T细胞密度与度伐利尤单抗的疗效相关，而活跃的APM及MHC表达，以及高CD4表达则与曲美木单抗的附加获益密切相关^[46]^。

在一项韩国真实世界的回顾性研究^[51]^中，接受化疗联合免疫治疗的SCLC-I亚型患者，不仅展现出最高的客观缓解率（objective response rate, ORR），其中位OS也呈现出优于其他亚型的趋势，尽管该差异未达统计学显著性。一项日本回顾性研究^[52]^证实，高密度的CD8^+ ^T细胞在TME中的浸润在组织层面反映了转录组定义的SCLC-I特征，能够显著预测阿替利珠单抗联合化疗的疗效，而传统的基于转录因子（ASCL1、NEUROD1、POU2F3）的病理亚型分型则与疗效无关。一项对接受一线化疗联合免疫治疗的中国ES-SCLC患者的回顾性研究^[43]^显示，SCLC-I亚型患者的中位PFS和OS均明显优于SCLC-A、SCLC-N及SCLC-P亚型；同时，SCLC-I亚型呈现出更高的CD8^+^ T细胞浸润和更活跃的免疫表型。这些结果提示SCLC-I亚型有望成为预测免疫治疗获益的潜在生物标志物。

**专家共识9：SCLC-I亚型与ES-SCLC患者ICIs联合化疗的高反应率及长期生存获益相关，可作为免疫治疗获益人群分层评估的参考指标（证据等级：B，推荐强度：强）。**

**专家共识10：SCLC-I亚型患者推荐采用免疫治疗联合化疗一线标准治疗（证据等级：A，推荐强度：强）；可进一步结合CD8** ^**+**^ **T细胞浸润、抗原递呈分子及髓系免疫微环境特征，筛选更易获得长期生存获益的人群（证据等级：C，推荐强度：强）。**

### 5.2 SCLC-A

SCLC-A亚型患者虽能从ICIs联合化疗中获益，但其疗效通常有限，现阶段其一线治疗仍应以免疫治疗联合化疗为基础。Delta样配体3（delta-like ligand 3, DLL3）作为ASCL1的关键转录靶点和NOTCH抑制性配体，是驱动SCLC NE分化的核心分子，也因此成为该亚型最具潜力的精准治疗靶点^[53]^。

靶向DLL3/CD3的双特异性T细胞衔接抗体（bispecific T-cell engager, BiTE）塔拉妥单抗在此领域取得突破性进展。在II期DeLLphi-301研究^[54]^中发现10 mg塔拉妥单抗在经治ES-SCLC患者中展现出较好疗效，ORR达40%，中位OS延长至14.3个月，并已获美国FDA加速批准，但目前国内尚未获批上市，相关临床研究正在开展中。作用机制上，塔拉妥单抗不仅能引导T细胞靶向杀伤肿瘤，临床前研究^[55]^还证实其可促进T细胞活化并上调PD-1/PD-L1表达，这为与ICIs的联合使用提供了理论依据。II期DeLLphi-303研究^[56]^的中期结果验证了这一潜力，显示塔拉妥单抗联合PD-L1抑制剂作为一线免疫治疗联合化疗后的维持治疗，中位OS达25.3个月，显著优于IMpower133研究的标准疗法（12.3个月）。该联合方案能否重塑一线治疗格局，正在III期DeLLphi-312研究中进行最终验证。此外，其他DLL3靶向药物（如Obrixtamig）的研发尚处于早期探索阶段，也为未来治疗提供了更多选择^[57]^。

近期的一项研究^[58]^阐明，NOTCH1是决定NE亚型（SCLC-A和SCLC-N）SCLC能否从一线免疫治疗联合化疗中获益的关键分子开关。通过对IMpower133试验数据的分析，研究发现，在NOTCH1高表达的SCLC-A亚型患者中，阿替利珠单抗联合化疗带来了显著的生存获益，中位OS延长至16.4个月，而对照组仅为8.3个月，死亡风险显著降低61%（HR=0.39, P=0.0012）；相比之下，NOTCH1低表达的患者则未能从免疫治疗中获益。机制探索发现NOTCH1能通过表观遗传方式重新激活STING信号通路，进而上调肿瘤细胞的抗原呈递能力，将TME从“免疫排斥”状态重编程为“免疫炎症”状态，从而有效逆转SCLC的免疫抑制。该研究不仅表明NOTCH1是一个具有潜力的预测性生物标志物，也为通过药理激活NOTCH1来增强免疫治疗疗效的联合策略提供了理论依据。

此外，基于SCLC-A亚型的生物学行为，其他靶向策略也展现出应用前景。靶向表观遗传调控因子EZH2和赖氨酸特异性脱甲基酶1（lysine-specific demethylase 1, LSD1）^[59,60]^可通过诱导肿瘤细胞向non-NE表型转换，从而逆转免疫抑制微环境并增敏免疫治疗；靶向凋亡通路的BCL-2抑制剂^[61]^及应用靶向B7同源物3（B7 homolog 3, B7-H3）^[62]^或滋养层细胞表面抗原2（trophoblast cell surface antigen 2, TROP2）^[63]^的抗体药物偶联物（antibody-drug conjugate, ADC），也为该亚型患者提供了更多潜在的治疗选择。

**专家共识11：SCLC-A亚型患者若无免疫治疗禁忌，目前仍推荐免疫治疗联合化疗作为一线基础治疗（证据等级：A，推荐强度：强）。**

**专家共识12：DLL3靶向治疗，尤其是DLL3/CD3双特异性T细胞衔接抗体等新型药物，是SCLC-A亚型患者治疗优化的重要探索方向，其在后线治疗、维持治疗及一线联合治疗中的临床价值值得进一步关注（证据等级：B，推荐强度：强）。**

**专家共识13：对于SCLC-A亚型，NOTCH1及相关表观遗传、凋亡通路靶点可用于联合治疗探索与优势人群筛选，但现阶段尚不宜单独作为临床治疗决策依据（证据等级：C，推荐强度：强）。**

### 5.3 SCLC-N

SCLC-N亚型常伴有MYC通路的异常激活，整体上更具侵袭性。既往研究提示，NEUROD1高表达与肿瘤细胞迁移、转移潜能增强有关。与SCLC-A亚型类似，SCLC-N对当前ICIs联合化疗的反应亦不理想。但就当前临床实践而言，现有证据尚不支持仅因SCLC-N分型而降低免疫治疗优先级。在IMpower133相关探索性分析^[12]^中，SCLC-A和SCLC-N亚型均可从一线免疫治疗联合化疗中获益，只是总体上获益幅度不及炎症相关亚型。

从治疗策略来看，SCLC-N亚型当前仍应以一线免疫治疗联合化疗为基础，但在经治患者和临床研究场景下，更值得优先关注MYC/AURKA轴及NEUROD1转录依赖相关治疗方向。研究^[12]^表明，SCLC-N亚型细胞对AURKA抑制剂敏感。其机制在于，MYC驱动的复制应激使细胞依赖于AURKA和CHK1以维持基因组稳定，抑制AURKA可导致细胞有丝分裂灾难和凋亡。一项在复发/难治性SCLC中开展的II期临床研究事后分析^[64]^显示，在c-Myc高表达的SCLC患者中，接受AURKA抑制剂阿利塞替联合紫杉醇治疗，相较于对照组显示出更优的PFS趋势。总体而言，AURKA以及NOTCH1/STING相关通路可作为SCLC-N亚型后续联合治疗和优势人群筛选的重要研究方向，但现阶段仍缺乏足以直接指导常规临床用药的前瞻性证据。

**专家共识14：SCLC-N亚型患者若无免疫治疗禁忌，目前仍推荐一线免疫治疗联合化疗作为基础治疗，不宜仅依据NEUROD1高表达而排除免疫治疗（证据等级：A，推荐强度：强）。**

**专家共识15：SCLC-N亚型中，靶向MYC/AURKA轴、NOTCH1通路及相关免疫增敏策略，可作为联合治疗探索与优势人群筛选的重要方向，现阶段不宜单独作为临床治疗决策依据（证据等级：C，推荐强度：强）。**

### 5.4 SCLC-P

SCLC-P亚型以POU2F3高表达为主要特征，通常缺乏经典的NE标志物，代表一类具有簇细胞样特征的non-NE型SCLC。现有研究^[12]^提示，该亚型在TIME构成和治疗敏感性方面与其他亚型存在差异，但其对ICIs联合化疗的获益模式尚未完全明确，部分研究甚至提示其整体预后可能相对较差。因此，SCLC-P亚型更重要的临床意义，可能在于识别其潜在的特异性治疗脆弱性，而非简单将其归类为免疫治疗低敏感人群。

已有临床前研究^[65,66]^表明，SCLC-P亚型可能对DNA损伤应答相关通路具有一定依赖性，尤其是聚腺苷二磷酸核糖聚合酶（poly(ADP-ribose) polymerase, PARP）相关机制，因此PARP抑制剂被视为该亚型值得关注的潜在治疗方向之一。一项I/II期试验研究数据^[65]^显示奥拉帕利联合替莫唑胺在经治ES-SCLC患者中显示出显著的抗肿瘤活性，ORR达41.7%，中位OS为8.5个月，为后线治疗提供了有效方案。一项单中心探索性临床研究^[66]^显示奥拉帕利联合帕博利珠单抗作为二线治疗，相较于帕博利珠单抗单药，显著改善了PFS，并展现出更高的ORR趋势。然而，奥拉帕利联合度伐利尤单抗的研究未达到预设有效性终点，而他拉唑帕利联合阿替利珠单抗维持治疗虽可改善PFS，但尚未带来明确的OS获益^[67,68]^。此外，研究^[30]^发现胰岛素样生长因子1受体（insulin-like growth factor 1 receptor, IGF-1R）在SCLC-P亚型中起关键作用，I期临床研究^[69]^显示IGF-1R单抗Dalotuzumab联合EP在ES-SCLC中的ORR为67%；然而IGF-1R抑制剂Linsitinib对比拓扑替康用于复发SCLC的II期临床研究则未达到主要终点^[70]^。这些结果提示，PARP抑制剂、IGF-1R靶向治疗在SCLC-P亚型中具有一定探索价值，但其获益人群尚不明确，目前相关研究仍处于临床试验探索阶段，国内外均尚未获批用于SCLC治疗，其临床价值有待进一步验证。因此对于SCLC-P亚型而言，PARP、IGF-1R相关治疗策略目前更适合作为后续临床研究和联合治疗优化的重要方向，尚不宜作为常规临床决策的依据。

**专家共识16：对于SCLC-P亚型患者，现阶段仍推荐以标准一线免疫治疗联合化疗为基础治疗策略（证据等级：A，推荐强度：强）；PARP抑制剂及IGF-1R靶向治疗等新型策略可用于联合治疗探索和优势人群筛选，但尚不宜单独作为临床治疗决策依据（证据等级：C，推荐强度：强）。**

## 6 ES-SCLC分子分型的动态监测

SCLC具有高度的异质性与亚型可塑性，表现为不同分子亚型和免疫特征的细胞亚群共存。这种异质性不仅存在于初治肿瘤，在治疗过程中也会发生动态演变^[34]^。表观遗传调控在此过程中发挥关键作用。抑制EZH2^[60]^可扩增具固有免疫信号的低NE细胞亚群；KDM1A（LSD1）抑制^[71]^通过恢复MHC-I表达及抗原呈递相关基因转录，激活干扰素信号并增强T细胞免疫应答；AURKA抑制^[72]^通过诱导肿瘤细胞阻滞于有丝分裂期，下调ASCL1并上调干扰素及抗原呈递相关基因。上述机制可促进高NE状态向MHC-I高表达、富含炎症特征的低NE状态（类似SCLC-I）转换，从而增强免疫治疗敏感性。KDM6A失活通过降低H3K4me1、升高H3K27me3重塑染色质状态，解除ASCL1驱动的转录程序，从而驱动ASCL1向NEUROD1的亚型转换^[73]^。在非表观遗传调控方面，MYC的激活可启动从SCLC-A到SCLC-N并最终向YAP1高表达状态的时序性演变^[32]^。临床前研究^[12]^显示，在以SCLC-A亚型为主的循环肿瘤细胞来源异种移植瘤模型中，顺铂治疗复发后出现了SCLC-I亚型细胞群的显著富集。临床回顾性分析^[49]^也证实，部分患者在疾病进展时分子亚型从SCLC-A转换为SCLC-I。

此外，亚型转换可能与肿瘤转移过程有关。研究^[74]^发现，在纵隔淋巴结转移灶中，部分原发灶为低NE的肿瘤产生了高NE的转移灶，提示NE分化可能增强肿瘤细胞的侵袭与转移能力。在脑转移灶中，仅有1/3的病例与原发灶亚型一致^[75]^。值得注意的是，动态异质性不仅存在于亚型层面，在DLL3、AURKA、PARP1等多个潜在治疗靶点的表达中也同样存在^[76]^。

为应对SCLC的异质性及治疗过程中的亚型演变，建议采用整合性动态监测策略，以精准指导临床决策。组织活检是诊断、分子分型以及TIME评估的金标准，能提供较完整的肿瘤信息。然而，作为侵入性操作，重复实施面临临床可行性与患者安全性的限制，且单点取材可能无法充分反映肿瘤内部的异质性。推荐在诊断初期与首次疾病进展时优先考虑组织活检。当临床或影像学提示肿瘤表型可能发生转变时，再次活检具有重要价值。液体活检为实现无创动态监测提供了有效手段。回顾性研究^[49]^表明，通过检测cfDNA的甲基化特征谱可对SCLC进行血浆分子分型，且该cfDNA甲基化图谱能够在疾病进展过程中识别亚型转换。

**专家共识17：鉴于ES-SCLC存在显著的分子异质性、亚型可塑性及治疗中动态演变，推荐在具备条件的临床实践或前瞻性临床研究中，采用以组织活检为基础、液体活检为补充的动态监测策略，为临床试验筛选及个体化综合治疗策略提供参考（证据等级：C，推荐强度：强）。**

## 7 总结

ES-SCLC的诊疗已进入免疫治疗时代，免疫治疗的应用为部分患者带来了明显生存获益，但整体疗效仍不理想，长期生存者比例有限，意味着目前的治疗模式尚无法充分应对该疾病高度异质的生物学特征。在这一背景下，分子分型为重新认识ES-SCLC的生物学本质、解释不同患者之间的疗效差异以及探索更精准的治疗策略提供了重要基础。但需要指出的是，分子分型在临床中的应用目前仍处于由研究走向转化的早期阶段，其真正融入常规诊疗流程仍面临诸多挑战。

未来的研究重点将集中在以下几个方面。首先，亟需更多前瞻性证据验证SCLC-A、SCLC-N、SCLC-P和SCLC-I不同亚型在一线免疫治疗联合化疗、维持治疗以及后线治疗中的敏感性差异，并明确哪些亚型或亚型相关生物学特征能够真正转化为具有临床指导意义的预测标志物。其次，分型检测体系本身仍需进一步规范。当前不同研究所采用的检测平台、判定方法、阳性阈值及评价标准并不一致，这在很大程度上限制了分子分型的可重复性和临床推广价值。尤其在病理学层面仍需统一IHC抗体克隆选择、标本处理流程、判读标准和报告模板，以提高不同中心之间检测结果的可比性。在治疗策略层面，联合治疗仍是未来改善预后的关键方向。DLL3靶向双抗、PARP抑制剂、表观遗传调控药物、免疫调节剂等新药物不断涌现，但如何结合分子分型及TIME特征筛选优势人群、确定最合适的联合模式，仍需进一步验证。尤其值得重视的是，利用表观遗传调控改善抗原呈递能力、重塑TIME并提高免疫治疗敏感性，是当前值得重视的研究方向之一。此外如何识别和应对治疗过程中出现的亚型转换，是临床管理的新挑战。越来越多证据显示，SCLC的分子特征并非静态，而是可能在化疗或免疫治疗压力下发生转变，从而影响后续疗效。因此未来需要将组织活检、cfDNA甲基化、转录组学等技术纳入动态监测体系，以便及时发现肿瘤表型变化，并为后续治疗调整提供依据。

基于上述论据与共识意见，本共识提出基于分子分型的ES-SCLC免疫治疗分层管理临床实施路径，以期为临床检测、研究分层、疗效预测、联合治疗策略探索及动态评估提供参考（图2）。总体而言，基于分子分型的精准免疫治疗体系仍处于构建阶段，但其潜力已经显现。随着分型体系不断优化、检测标准逐步统一、动态监测手段日益成熟以及更多前瞻性研究证据的积累，未来有望逐步建立更具针对性的精准诊疗路径，从而进一步改善ES-SCLC患者的整体预后。

**图 2:**
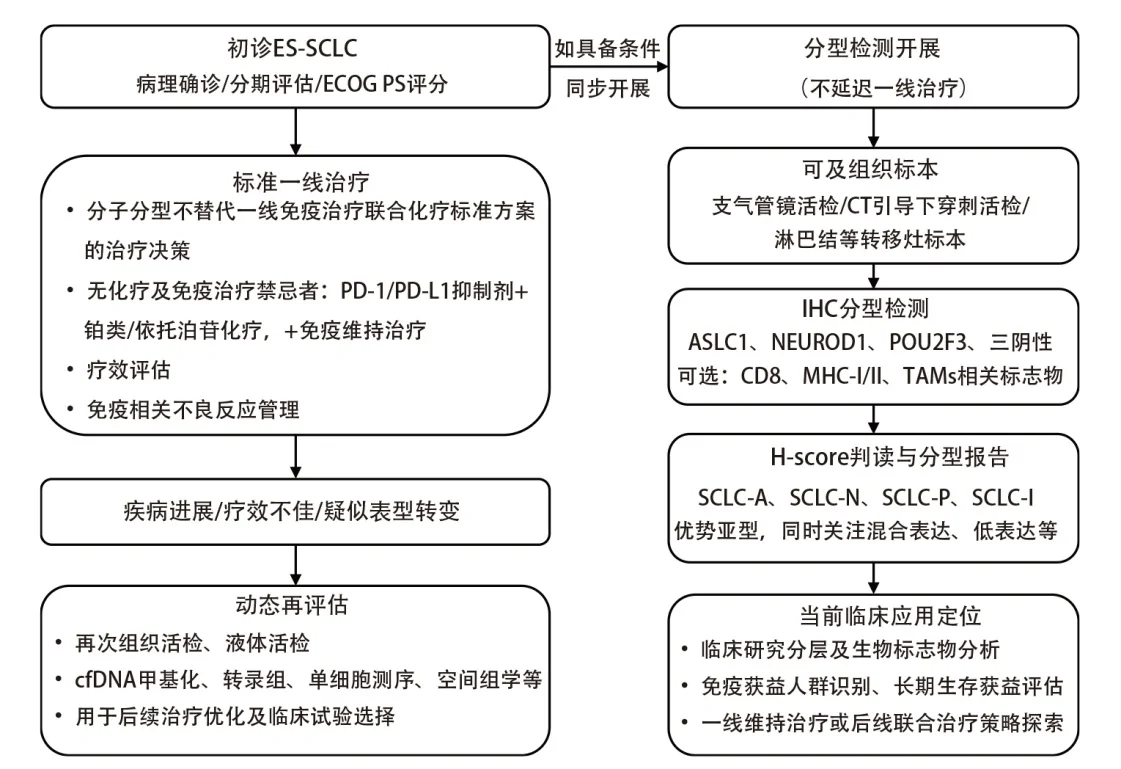
基于分子分型的ES-SCLC免疫治疗分层管理与动态评估流程图 cfDNA：循环游离DNA；ECOG PS：美国东部肿瘤协作组体能状态；IHC：免疫组化；CT：计算机断层扫描。

## 共识编写组成员

**组长：**周建娅（浙江大学医学院附属第一医院）

**执笔专家：**屈晶晶（浙江大学医学院附属第一医院），吴琳颖（浙江大学医学院附属第一医院）

**专家组成员**（按姓氏汉语拼音字母排序）：储天晴（上海交通大学医学院附属胸科医院），段建春（中国医学科学院肿瘤医院），黄岩（中山大学肿瘤防治中心），李玉苹（温州医科大学附属第一医院），林冬梅（北京大学肿瘤医院），柳菁菁（吉林省肿瘤医院），吕冬青（浙江省台州医院），吕镗烽（东部战区总医院），孟祥姣（山东第一医科大学附属肿瘤医院），屈晶晶（浙江大学医学院附属第一医院），饶创宙（宁波市第二医院），生万强（浙江大学医学院），苏春霞（上海市肺科医院），滕晓东（浙江大学医学院附属第一医院），涂海燕（广东省人民医院），吴琳颖（浙江大学医学院附属第一医院），项轶（上海交通大学医学院附属瑞金医院），杨农（湖南省第二人民医院），于壮（青岛大学附属医院），张健（南方医科大学珠江医院），周建娅（浙江大学医学院附属第一医院），朱艳丽（北京大学肿瘤医院）

**特邀顾问：**周建英（浙江大学医学院附属第一医院），张绪超（广东省人民医院）
